# Increased Expression of Intranuclear Matrix Metalloproteinase 9 in Atrophic Renal Tubules Is Associated with Renal Fibrosis

**DOI:** 10.1371/journal.pone.0048164

**Published:** 2012-10-24

**Authors:** Jen-Pi Tsai, Jia-Hung Liou, Wei-Tse Kao, Shao-Chung Wang, Jong-Da Lian, Horng-Rong Chang

**Affiliations:** 1 Institute of Medicine, Chung Shan Medical University, Taichung, Taiwan; 2 Department of Nephrology, Buddhist Dalin Tzu Chi General Hospital, Chiayi, Taiwan; 3 Department of Pathology, Changhua Christian Hospital, Changhua, Taiwan; 4 Department of Medical Technology, Jen-The Junior College of Medicine, Nursing and Management, Miaoli, Taiwan; 5 Department of Urology, Chung Shan Medical University Hospital, Taichung, Taiwan; 6 Division of Nephrology, Department of Internal Medicine, Chung Shan Medical University Hospital, Taichung, Taiwan; University of Patras, Greece

## Abstract

**Background:**

Reduced turnover of extracellular matrix has a role in renal fibrosis. Matrix metalloproteinases (MMPs) is associated with many glomerular diseases, but the histological association of MMPs and human renal fibrosis is unclear.

**Methods:**

This is a retrospective study. Institutional Review Board approval was obtained for the review of patients’ medical records, data analysis and pathological specimens staining with waiver of informed consents. Specimens of forty-six patients were examined by immunohistochemical stain of MMP-9 in nephrectomized kidneys, and the association of renal expression of MMP-9 and renal fibrosis was determined. MMP-9 expression in individual renal components and fibrosis was graded as high or low based on MMP-9 staining and fibrotic scores.

**Results:**

Patients with high interstitial fibrosis scores (IFS) and glomerular fibrosis scores (GFS) had significantly higher serum creatinine, lower estimated glomerular filtration rate (eGFR), and were more likely to have chronic kidney disease (CKD) and urothelial cell carcinoma. Univariate analysis showed that IFS and GFS were negatively associated with normal and atrophic tubular cytoplasmic MMP-9 expression and IFS was positively correlated with atrophic tubular nuclear MMP-9 expression. Multivariate stepwise regression indicated that MMP-9 expression in atrophic tubular nuclei (r = 0.4, p = 0.002) was an independent predictor of IFS, and that MMP-9 expression in normal tubular cytoplasm (r = −0.465, p<0.001) was an independent predictor of GFS.

**Conclusions:**

Interstitial fibrosis correlated with MMP-9 expression in the atrophic tubular nuclei. Our results indicate that renal fibrosis is associated with a decline of MMP-9 expression in the cytoplasm of normal tubular cells and increased expression of MMP-9 in the nuclei of tubular atrophic renal tubules.

## Introduction

Scarring of renal tissue, which occurs in glomerulosclerosis, interstitial fibrosis, and tubular atrophy, is caused by a variety of primary insults, such as diabetes mellitus (DM), hypertension (HTN), primary glomerulopathies, autoimmune diseases, toxic injury, and congenital abnormalities [1]. The pathogenesis of renal fibrosis includes deposition of interstitial matrix, tubular cell loss, infiltration of inflammatory cells, fibroblast accumulation, rarefaction of peritubular microvasculature, and predisposition to renal progression in the presence of genetic polymorphisms [2]. Extracellular matrix (ECM) components accumulate during renal fibrosis resulting from an imbalance of ECM production and defective ECM degradation by proteolytic enzymes. Matrix metalloproteinases (MMPs) play a major role in ECM degradation.

MMPs are a family of zinc-dependent endopeptidases that are currently divided into six groups with varying substrate specificities modulated by tissue inhibitors of metalloproteinases (TIMPs). MMPs work synergistically to degrade ECM components and are involved in a variety of pathophysiological processes in which tissue remodeling is needed, such as embryonic development, angiogenesis, invasive cell behavior, inflammation, wound healing, and fibrosis [3], [4]. Kidney tissue produces a number of proteases, and the MMP system and plasminogen/plasmin play major roles in degrading matrix proteins [3], [5]. Changes in expression or activity of MMPs alter ECM turnover, and this can lead to glomerular sclerosis and other glomerulonephropathies (GNs) [6], [7], [8], [9].

MMP-9 (gelatinase B), a 92 kDa type IV collagenase, is regulated through formation of proenzyme complexes with endogenous TIMP-1. MMP-9 can specifically degrade type IV and V collagens and gelatine [3]. The spatial expression of MMP-9 in the kidney is complex and species-specific. MMP-9 is mainly expressed in collecting duct cells and to a lesser extent in proximal tubule and podocytes of mice [10], in the proximal and distal tubules of monkeys [11], and in glomerular mesangial cells of humans [12]. MMP-9 is initially believed to be involved in the pathogenesis of chronic kidney disease (CKD). We recently reported that the circulating level of MMP-9 was inversely correlated with serum creatinine (r = −0.344, p<0.01) [13].

**Table 1:** Demographic and clinical characteristics of patients divided by low and high renal interstitial fibrosis score (left) and glomerular fibrosis score (right).

	Fibrosis score (interstitium)			Fibrosis score (glomerulus)		
	low	high	P value	low	High	P value
Patient Number	28	18		34	12	
Gender (Male, %) Age (years)	13 (46.4) 56.1±14.8	10 (55.6) 60.2±10.3	0.546 0.431	17 (50) 57.2±14.6	6 (50) 59.2±8.8	1 0.783
CKD (n, %)	10 (35.7)	13 (72.2)	0.016	13 (38.2)	10 (83.3)	0.007
eGFR (ml/min)	74.2±22.7	42.2±27.5	0.001	72.2±22.3	32.1±25.8	<0.001
Creatinine (mg/dl)	1.04±0.32	3.59±3.93	0.001	1.07±0.32	4.78±4.38	<0.001
DM (n, %)	5 (18.5)	4 (22.2)	0.761	7 (21.2)	2 (16.7)	0.736
HTN (n, %)	10 (35.7)	10 (55.6)	0.185	12 (35.3)	8 (66.7)	0.059
BMI (kg/m^2^)	24.5±3.8	25.3±3.2	0.266	24.9±4	24.6±2.1	0.729
Smoker (n, %)	3 (11.1)	1 (5.6)	0.521	4 (12.1)	0 (0)	0.206
Glucose (mg/dl)	111.8±27.7	142.4±67.9	0.223	114.6±33.8	151.9±75.5	0.134
Hemoglobin (g/dl)	10.6±3.57	10.1±2.8	0.398	10.7±3.5	9.6±2.3	0.087
Albumin (mg/dl)	3.79±0.92	4.01±0.39	0.897	3.88±0.87	3.9±0.36	0.345
TCH (mg/dl)	176.6±32.1	195.9±49	0.18	181.6±36.4	199.8±54.5	0.432
Triglyceride (mg/dl)	143.0±75.5	165.6±84.6	0.487	146.8±82.9	175.9±72.9	0.485
Diagnosis (n, %)						
UCC	4 (14.3)	10 (55.6)	<0.001	8 (23.5)	6 (50)	<0.001
RCC	20 (71.4)	2 (11.1)		22 (64.7)	0 (0)	
Other	4 (14.3)	6 (33.3)		4 (11.8)	6 (50)	

BMI, body mass index; CKD, chronic kidney disease; DM, diabetes mellitus; eGFR, estimated glomerular filtration; HTN, hypertension; RCC, renal cell carcinoma; UCC, urothelial cell carcinoma, TCH, total cholesterol.

*p*<0.05 indicates significance.

Based on these previous studies and because MMP-9 is associated with ECM accumulation and tubulointerstitial fibrosis, we examined the relationship between histological renal expression of MMP-9 and renal fibrosis, including glomerular and interstitial fibrosis. We used human renal tissues which had various degrees of fibrosis that were remnants from previously nephrectomized kidneys.

**Table 2:** Intensity of MMP-9 expression in different regions of renal tissues divided by low and high interstitial fibrosis score (left) and glomerular fibrosis score (right).

	Fibrosis score (interstitium)			Fibrosis score (glomerulus)		
	low	high	P value	low	high	P value
MMP-9 intensity						
NTn (n, %)						
Low	26 (92.9)	18 (100)	0.246	32 (94.1)	12 (100)	0.39
High	2 (7.1)	0 (0)		2 (5.9)	0 (0)	
NTc (n, %)						
Low	1 (3.6)	7 (38.9)	0.002	1 (2.9)	7 (58.3)	<0.001
High	27 (96.4)	11 (61.1)		33 (97.1)	5 (41.7)	
Gn (n, %)						
Low	28 (100)	18 (100)		34 (100)	12 (100)	
High						
Gc (n, %)						
Low	16 (57.1)	14 (77.8)	0.152	20 (58.8)	10 (83.3)	0.125
High	12 (42.9)	4 (22.2)		14 (41.2)	2 (16.7)	
ATn (n, %)						
Low	25 (89.3)	11 (61.1)	0.024	28 (82.4)	8 (66.7)	0.257
High	3 (10.7)	7 (38.9)		6 (17.6)	4 (33.3)	
ATc (n, %)						
Low	8 (28.6)	12 (70.6)	0.006	12 (35.3)	8 (72.7)	0.03
High	20 (71.4)	5 (29.4)		22 (64.7)	3 (27.3)	

NTn, normal tubular nucleus; NTc, normal tubular cytoplasm; Gn, glomerular nuclei; Gc, glomerular cytoplasm; ATn, atrophic tubular nuclei; ATc, atrophic tubular cytoplasm.

Data were analyzed by the chi-squared test and *p*<0.05 indicates significance.

## Materials and Methods

From January 2006 to August 2009, pathological specimens from 90 patients who received unilateral or bilateral nephrectomy were retrospectively recruited. Institutional review board approval of Chung Shan Medical University Hospital was obtained for the review of patients’ medical records, data analysis and pathological specimens staining with waiver of informed consents.

**Table 3:** Associations between interstitial and glomerular fibrosis with clinicopathologic variables.

	Interstitial fibrosis		Glomerular fibrosis	
Variable	Beta	P value	Beta	P value
Age (year)	0.150	0.319	0.070	0.644
Sex (male, %))	0.089	0.556	0.000	1
Chronic kidney disease (n, %)	0.356	0.015	0.396	0.006
Creatinine (mg/dl)	0.461	0.001	0.603	<0.001
Estimated GFR (ml/min)	−0.544	<0.001	−0.612	<0.001
Body mass index (kg/m^2^)	0.009	0.436	−0.043	0.780
Smoker (n, %)	−0.096	0.532	−0.188	0.215
Diabetes mellitus (n, %)	0.045	0.767	−0.050	0.743
Hypertension (n, %)	0.195	0.193	0.278	0.061
Pathologic diagnosis	0.489	0.001	0.571	<0.001
Glucose (mg/dl)	0.302	0.062	0.333	0.038
Hemoglobin (g/dl)	−0.09	0.611	−0.161	0.362
Albumin (mg/dl)	0.157	0.444	0.012 0.012	0.954
Total cholesterol (mg/dl)	0.230	0.269	0.202	0.332
Triglyceride (mg/dl)	0.144	0.501	0.170	0.428
MMP-9 intensity (n, %)				
Glomerular cytoplasm	−0.211	0.158	−0.226	0.131
Atrophic tubular nucleus	0.333	0.024	0.167	0.267
Atrophic tubular cytoplasm	−0.410	0.005	−0.324	0.030
Normal tubular nucleus	−0.171	0.256	−0.127	0.402
Normal tubular cytoplasm	−0.455	0.001	−0.642	<0.001

Interstitial fibrosis scores were significantly correlated with glomerular fibrosis scores (r = 0.741, *p*<0.001).

*p*<0.05 was considered statistically significant.

Of this study, forty-six patients who had stable renal function for more than 3 months before surgery were ultimately included. Patient age, gender, body mass index, status of cigarette smoking, HTN, and DM were recorded. Estimated glomerular filtration rate (eGFR) was calculated by the abbreviated Modification of Diet in Renal Disease formula (aMDRD):
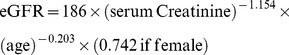



Chronic kidney disease (CKD) was defined by K/DOQI guidelines [14].

**Figure 1:**
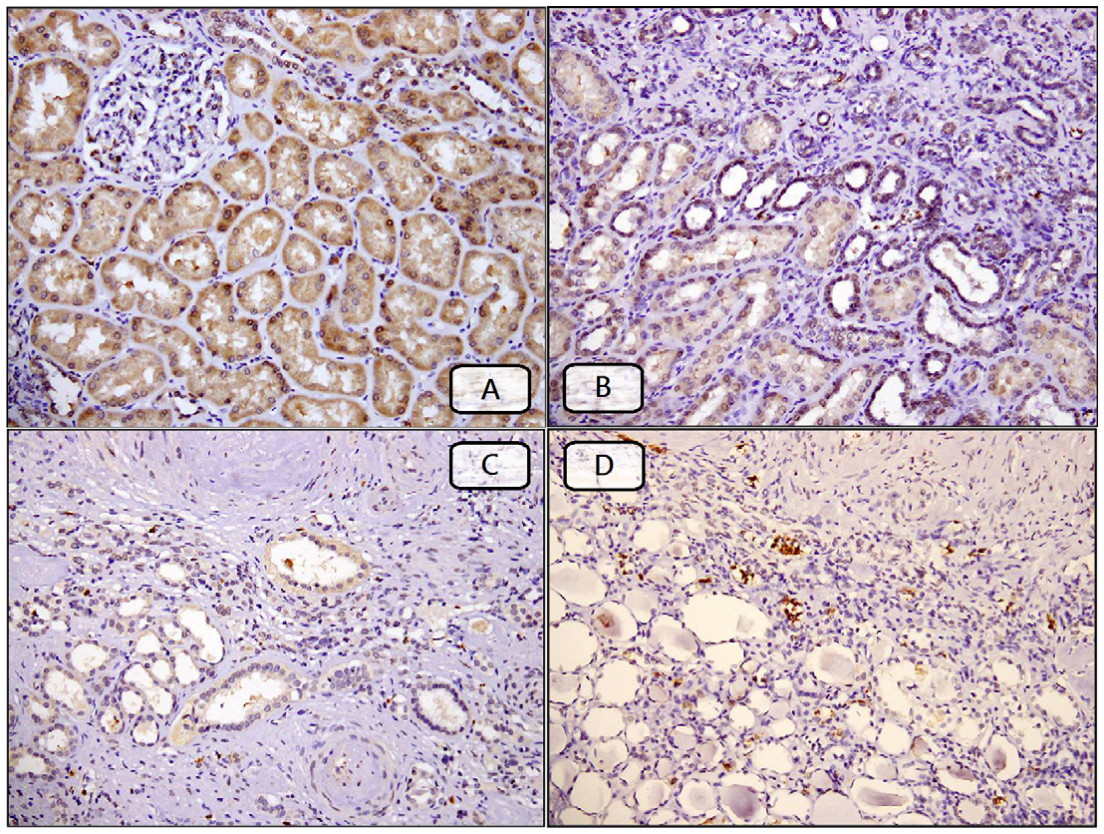
Representative panels showing different expression intensity of MMP-9 in atrophic tubular nuclear compared to normal tubular cytoplasm nearby fibrotic renal parenchyma. (A) no fibrosis with increased cytoplasm stain in normal tubules, (B) mild fibrosis with decreased cytoplasm stain in normal tubules and increased nuclear stain in atrophy tubules, (C) severe fibrosis with decreased cytoplasm stain in normal tubules, and (D) severe fibrosis with increased nuclear stain in atrophy tubules. (IHC stain, x 20).

**Table 4:** Multivariate analysis with stepwise linear regression of factors independently associated with interstitial and glomerular fibrosis.

	Interstitial fibrosis		Glomerular fibrosis	
Variable	Beta	P value	Beta	P value
Pathological diagnosis (n, %)	0.656	<0.001	0.511	<0.001
MMP-9 intensity (n, %)				
Atrophic tubular nucleus	0.400	0.002		
Normal tubular cytoplasm			−0.465	<0.001

Variables included p less than 0.1 in univariate linear regression analysis.

*p*<0.05 was considered statistically significant.

### Tissue processing

Pathologic material was processed by conventional histological procedures. Representative sections were taken in the renal parenchyma at least 2 cm away from the tumor areas in cases with nephrectomy due to tumor. Each section was at least 2×2 cm^2^. The formalin-fixed, paraffin-embedded tissues were cut into 4-mm hematoxylin- and eosin-stained sections and examined to evaluate the glomerular, renal tubular, and interstitial conditions. The scoring of fibrosis was based on Banff scoring for chronic lesions [15]. The low fibrosis group was defined by a score of 0 or 1 in the interstitium and glomeruli, and the high fibrosis group was defined by a score of 2 or 3 in these tissues.

**Table 5:** Expression of MMP-9 in different renal tissues between patients with and without cancer.

	Cancer	Non-cancer	P value
MMP-9 intensity			
NTn (n, %)			1.0
Low	34 (94.4)	10 (100)	
High	2 (5.6)	0 (0)	
NTc (n, %)			0.344
Low	5 (13.9)	3 (30)	
High	31 (86.1)	7 (70)	
Gc (n, %)			0.72
Low	24 (66.7)	6 (60)	
High	12 (33.3)	4 (40)	
ATn (n, %)			0.089
Low	26 (72.2)	10 (100)	
High	10 (27.8)	0 (0)	
ATc (n, %)			0.083
Low	13 (37.1)	7 (70)	
High	22 (62.9)	3 (30)	

Abbreviation: Gc, glomerular cytoplasm; NTn, normal tubular nucleus; NTc, normal tubular cytoplasm; ATn, atrophic tubular nuclei; ATc, atrophic tubular cytoplasm.

Data were analyzed by the chi-squared test and Fisher’s exact test accordingly and *p*<0.05 indicates significance.

**Table 6:** Associations between intensity of MMP-9 expression and cancer over different renal tissues.

	Cancer	
Variable	Beta	P value
MMP-9 intensity		
Glomerular cytoplasm	0.058	0.703
Atrophic tubular nucleus	−0.278	0.062
Atrophic tubular cytoplasm	−0.275	0.068
Normal tubular nucleus	−0.112	0.457
Normal tubular cytoplasm	−0.175	0.244

*p*<0.05 was considered statistically significant.

### Immunohistochemical staining

Paraffin embedded kidney tissue sections (4-mm) on poly-1-lysine-coated slides were deparaffinized. After treatment with 3% H_2_O_2_ in methanol, the sections were hydrated with gradient alcohol and PBS, incubated in 10 mM citrate buffer, and finally heated at 100°C for 20 min in PBS. Slides were incubated with the anti-MMP-9 antibody (Santa Cruz, CA) for 20 min at room temperature, and then with a horseradish peroxidase (HRP)/Fab polymer conjugate for another 30 min. Then, slides were thoroughly washed three times with PBS, and the sites of peroxidase activity were visualized using 3, 3-diamino-benzidine tetrahydrochloride as a substrate and hematoxylin as the counter stain. All immunohistochemical (IHC) data were independently scored by two blinded pathologists. Every slide was examined entirely for nuclear and cytoplasmic MMP-9 stains in the normal and atrophic renal tubules and in the normal and atrophic glomeruli. Each 2×2 cm^2^ section contained at least 30 glomerular areas, and the actual number of examined glomeruli was based on the sectioned tissue size. The number of immunoreactive cells was calculated semi-quantitatively and evaluated as a percentage (0∼100%) of positive cells in the observed tubules and glomeruli (normal and atrophic) as follows: intensity 0, negative; intensity 1+, 1∼10%; 2+, 10∼50%; and 3+, >50% [16]. The results of nuclear and cytoplasmic staining were recorded separately. The intensity of MMP-9 staining was classified as high (2 and 3) or low (0 and 1).

### Statistical analysis

Continuous and categorical data were expressed as means ± standard deviations and as proportions, respectively. Categorical variables were analyzed by the chi-square test. The statistical significance between continuous variables was analyzed by the Mann-Whitney U test. Correlations of clinical variables with interstitial and glomerular fibrosis were evaluated by univariate linear regression analysis. Variables with *p*-values less than 0.1 in the univariate linear regression analysis were used for stepwise multivariate linear regression analysis to analyze the independent association of interstitial and glomerular fibrosis with clinical and pathological variables. A *p*-value less than 0.05 was considered statistically significant. All data were analyzed using SPSS version 14.0 statistical software.

## Results

The mean age of the 46 patients at surgery was 57.7±13.2 years. Nine patients (19.6%) had DM, 20 patients (43.5%) had HTN, and 23 patients (50%) had CKD. Fourteen patients (30.4%) were given nephrectomies due to urothelial cell carcinoma (UCC) and 22 patients (47.8%) were given nephrectomies due to renal cell carcinoma (RCC).

We classified the 46 patients based on high or low scores for interstitial and glomerular fibrosis. In particular, we compared the association between the interstitial fibrosis score (IFS) and glomerular fibrosis score (GFS) with the intensity of MMP-9 expression in each component of the specimen if both the compared components were on the same specimen. Our results indicate that IFS was inversely associated with eGFR (42.2±27.5 mL/min for high IFS, 74.2±22.7 mL/min for low IFS, *p* = 0.001), positively associated with CKD (72.2% for high IFS, 35.7% for low IFS, *p* = 0.016), and positively associated with UCC (55.6% for high IFS, 14.3% for low IFS, *p*<0.001). Similarly, GFS was inversely associated with eGFR (32.1±25.8 mL/min for high GFS, 72.2±22.3 mL/min for low GFS, *p*<0.001), positively associated with CKD (83.3% for high GFS, 38.2% for low GFS, *p*<0.007), and positively associated with UCC (50% for high GFS, 23.5% for low GFS, *p*<0.001) (Table 1).

IFS was inversely associated with expression of MMP-9 in normal tubular cytoplasm (NTc) (61.1% for high IFS, 96.4% for low IFS, *p* = 0.002) and in atrophic tubular cytoplasm (ATc) (29.4% for high IFS, 71.4% for low IFS, *p* = 0.006), but positively associated with expression of MMP-9 in atrophic tubular nucleus (ATn) (38.9% for high IFS, 10.7% for low IFS, *p* = 0.024). GFS was inversely associated with expression of MMP-9 in normal tubular cytoplasm (NTc) (41.7% for high GFS, 97.1% for low GFS, *p*<0.001) and in atrophic tubular cytoplasm (ATc) (27.3% for high GFS, 64.7% for low GFS, *p* = 0.03) (Table 2).

Table 3 showed the association between IFS and GFS and clinical and histological variables. Univariate analysis indicated that IFS was positively associated with serum creatinine (r = 0.461, *p* = 0.001), presence of CKD (r = 0.356, *p* = 0.015), and MMP-9 intensity in ATn (r = 0.333; *p* = 0.024), and negatively associated with eGFR (r = −0.544, *p*<0.001), and MMP-9 expression in ATc (r = −0.410, *p* = 0.005) and NTc (r = −0.455, *p* = 0.001). GFS was positively associated with serum creatinine (r = 0.603, *p*<0.001), presence of CKD (r = 0.396, *p* = 0.006), and blood glucose (r = 0.333, *p* = 0.038), and negatively associated with eGFR (r = −0.612, *p*<0.001), and MMP-9 expression in ATc (r = −0.324, *p* = 0.03) and NTc (r = −0.642, *p*<0.001). IFS and GSF were each associated with the pathological diagnosis of the nephrectomised kidney(s) (r = 0.498, *p* = 0.001; r = 0.571, *p*<0.001, respectively). There was positive correlation between the expression of IFS and GFS (r = 0.741, *p*<0.001).

Finally, we employed stepwise multivariate linear regression to identify the independent predictors of IFS and GFS. All variables with *p*-values less than 0.1 in the univariate linear regression were included in this analysis (serum creatinine, status of HTN, fasting glucose, pathological diagnosis, and MMP-9 intensities in ATc, ATn and NTc). The results indicated that MMP-9 intensity in ATn (r = 0.40, *p* = 0.002) was an independent factor predicting IFS and that MMP-9 intensity in NTc (r = −0.465, *p*<0.001) was an independent factor predicting GFS (Table 4). Patients with different pathological diagnoses had significant correlation with IFS and GFS (r = 0.656, *p*<0.001; r = 0.511, *p*<0.001, respectively). Figure 1 showed a representative cross-section in which there was increased expression of MMP-9 in atrophic tubular nuclei (Panel B and D) and decreased in the normal tubular cytoplasm (Panel B and C) simultaneously.

In addition, we divided our patients into those without or with urinary tract cancers (included UCC and RCC) to evaluate the relationship between the intensity of MMP-9 expression and urinary tract cancers. Table 5 showed comparable percentage of MMP-9 expression over different renal tissues between groups. By univariate linear regression, there was no relationship between urinary tract cancer and intensity of MMP-9 expression over the renal tissues (Table 6).

## Discussion

Our results demonstrated that the extent of interstitial fibrosis was associated with the intensity of MMP-9 expression in atrophic tubular nuclei, and that the extent of glomerular fibrosis was inversely associated with MMP-9 expression in normal tubular cytoplasm. In other words, the process of renal fibrosis involves a decline of MMP-9 expression in normal tubular cytoplasm and an increased expression of MMP-9 in the tubular nuclei of atrophic renal tubules. Although the molecular basis of increased intranuclear MMP-9 expression in renal fibrosis is still unknown, these findings form a basis for further investigation of the role of MMP 9 in human renal injury.

Bengatta et al. reported that in a mouse model of acute kidney injury, MMP-9 expression was markedly increased in the S3 segment of the proximal tubule [17]. They postulated that MMP-9 had a protective role, because MMP-9 deficiency increased apoptosis and severity of renal lesions and substantially delayed recovery of renal function in their model. Previous studies of a rat model of tubulointerstitial fibrosis and glomerulosclerosis indicated reduced expression of MMP-9 [18], [19]. Moreover, a study of diabetic nephropathy in a rat model indicated decreased MMP-9 expression and activity (mRNA and enzymatic activity of MMP-9: 21% and 51% respectively, *p*<0.05 *vs*. control), compatible with the increased ECM deposition associated with this disease [20]. Taken together, these data suggested that ECM turnover, which was modulated by MMPs, increases in the presence of acute kidney injury, but reduced degradation of MMPs ultimately resulted in development of renal fibrosis. Wang et al had reported that MMP-9 could modulate renal interstitial fibrosis in obstructive nephropathy by blocking tubular epithelial-to-myofibroblast, preserving tubular basement membrane and reducing ECM expression [21]. An angiotensin converting enzyme inhibitor, ramipril, had been investigated to find the contribution of MMP-9 in the process of glomeruloscelrosis and chronic renal disease in hypertensive rats. MMP-9 mRNA expression was markedly suppressed to 10% of control levels independent of the treatment of ramipril, which suggested that the MMP-9 might play a role via other mechanisms other than inhibition of angiotensin converting enzyme inhibitor [19]. Similarly, our results with human tissue indicated decreased MMP-9 expression in NTc was associated with higher GFS. This indicated that the process of glomerulosclerosis involved intracellular degradation of cytosolic MMP-9.

Bauvois et al. evaluated the correlation between plasma MMP/TIMP expression and renal tissue fibrosis (glomerular sclerosis and interstitial fibrosis) in 83 patients [8]. They reported a relationship between the level of plasma MMP/TIMP and tissue fibrosis from these biopsy-proven cases of GN, but neither plasma MMPs nor TIMP-1 were significantly associated with risk of poor renal outcome (final serum creatinine <30 mL/min/1.73 m^2^). The only significant risk factors were baseline creatinine clearance (odds ratio, 0.97; 95% confidence interval 0.95–0.99; *p* = 0.0057) and interstitial fibrosis (odds ratio, 1.46; 95% confidence interval 1.01–2.14; *p* = 0.045).

In addition to cleavage of the extracellular matrix by MMPs, proteolysis of nuclear matrix was implicated in numerous other cellular processes, such as apoptosis, cell cycle regulation, and DNA fragmentation [22]. Yang et al. had investigated the relationship of intracellular MMP-9 with plasma level of MMP-9 and tissue damage [22]. They designed an ischemic-reperfusion rat model, with a 90 min middle artery occlusion, and also used tissue from stroke patients to investigate the role of MMP-9 in ischemic brain neurons. Their results indicated an association of increased intranuclear MMP-9 activity in ischemic neurons at 3 h and increased DNA fragmentation at 24 h and 48 h after reperfusion. Nuclear MMPs had been reported to modulate cellular process by cleavage of the nuclear matrix protein poly-ADP-ribose-polymerase (PARP), an ATP-dependent DNA repair enzyme, and to inactivate PARP in a time-dependent manner. This was similar to the role of caspase-3, which played a protective role when PARP was over-activated and a detrimental effect by hindering repair of DNA strand breaks [23]. MMP inhibition also mediated increased activity of PARP-1 and decreased level of oxidized DNA in ischemic brain cells. In particular, Yang et al. [22] proposed that the increased intranuclear MMP-9 activity soon after stroke degraded PARP-1 and X-ray cross-complementary factor 1, contributing to a reduction of DNA base excision repair and accumulation of oxidized DNA bases in neurons, triggering neuronal death. Similarly, we noted increased MMP-9 expression in ATn was associated with greater IFS (r = 0.40, *p* = 0.002). Our results indicated that increased nuclear expression of MMP-9 in human atrophic renal tubular cells may play a role in the process of renal injury or fibrosis, although the molecular mechanism may differ from that proposed by Yang et al. for ischemic brain injury [22].

Although we found that the expression of MMP-9 correlated with tubulointerstitial fibrosis, there were reports showing that the MMP-9 expressions could be affected by upper urothelium carcinogenesis [24] and RCC [25]. In addition, Gialeli et al. had reported that MMP-9 was capable to proteolytically modulate ECM which could promote tumor progression, and MMP inhibitors had been studied to control the enzyme activities to therapeutically intervene carcinogenesis [26]. Because38 out of our 46 patients who received nephrectomies were due to urinary tract cancers (UCC and RCC). To reduce the possible impact of urinary tract cancers on the MMP-9 expression, we conducted this study by taking specimens at least 2 cm from the tumors. In addition, to test whether urinary tract cancers were associated with the intensity of MMP-9 expression in the adjacent renal tissues, we divided our patients into those without or with urinary tract cancers. The results showed that there were no significant association between the intensity of MMP-9 expression and various parts of renal tissues. Therefore, we considered that the intensity of MMP-9 expression in this study was independent of urinary tract cancers.

In summary, our analysis of the spatial expression of MMP-9 in human nephrectomized specimens indicates a novel role for MMP-9 in renal fibrosis. We postulate that increased intranuclear MMP-9 expression may reflect intranuclear gelatinase proteolysis, play a role in oxidative DNA damage by cleaving nuclear matrix proteins (PARP-1 and/or XRCC1), and contribute to cell death and fibrosis. Further experiments are needed to support this postulated mechanism.
